# Endoscopic ultrasound-guided radiofrequency ablation for a pancreatic insulinoma: a novel endoscopic management strategy

**DOI:** 10.1055/a-2712-3773

**Published:** 2025-10-09

**Authors:** Khalid Ahmed, Rahul Karna, Mohammad Alutaibi, Jimmie Stewart III, Kidmealem Zekarias, Guru Trikudanathan

**Affiliations:** 1 311816 Department of Medicine, Division of Gastroenterology, Hepatology and Nutrition, University of Minnesota System, Minneapolis, United States; 2 311816 Department of Laboratory Medicine and Pathology, University of Minnesota System, Minneapolis, United States; 3 311816 Department of Medicine, Division of Diabetes, Endocrinology and Metabolism, University of Minnesota System, Minneapolis, United States

Although rare, insulinomas are the most common functional pancreatic neuroendocrine tumors (NETs), typically presenting with Whipple’s triad – symptoms of hypoglycemia, low plasma glucose, and symptom resolution after glucose administration 1 . While surgery remains the primary curative treatment for an insulinoma, its potential for significant adverse outcomes has prompted exploration of less invasive approaches, such as endoscopic ultrasound-guided radiofrequency ablation (EUS-RFA) 2 3 . EUS-RFA has shown high efficacy and a favorable safety profile in the treatment of insulinomas, especially for patients who are not suitable for surgery 4 . The purpose of presenting this case is to highlight the role of EUS-RFA as a minimally invasive and effective treatment option for an insulinoma in a patient who declined to undergo surgery ( Video 1 ).

Endoscopic ultrasound-guided radiofrequency ablation is performed to treat an insulinoma in the pancreatic head.Video 1

A 37-year-old man with Crohn’s disease presented with recurrent fasting hypoglycemia and met Whipple’s triad. Laboratory testing showed endogenous hyperinsulinemia. Computed tomography and positron emission tomography (PET) ^68^ GA-dotatate scans were negative. EUS revealed a 13-mm well-differentiated NET in the pancreatic head, with additional subcentimeter lesions, and fine-needle biopsy confirmed this was an insulinoma ( Fig. 1 ). Surgical enucleation was deemed high risk owing to proximity to the main pancreatic duct and the risk of fistula formation, and the patient declined to undergo a Whipple surgery. After multidisciplinary review, EUS-RFA was selected as the treatment modality. The procedure was technically successful without complications, with complete resolution of the patient’s symptoms and improvement of his biochemical parameters.

**Fig. 1:**
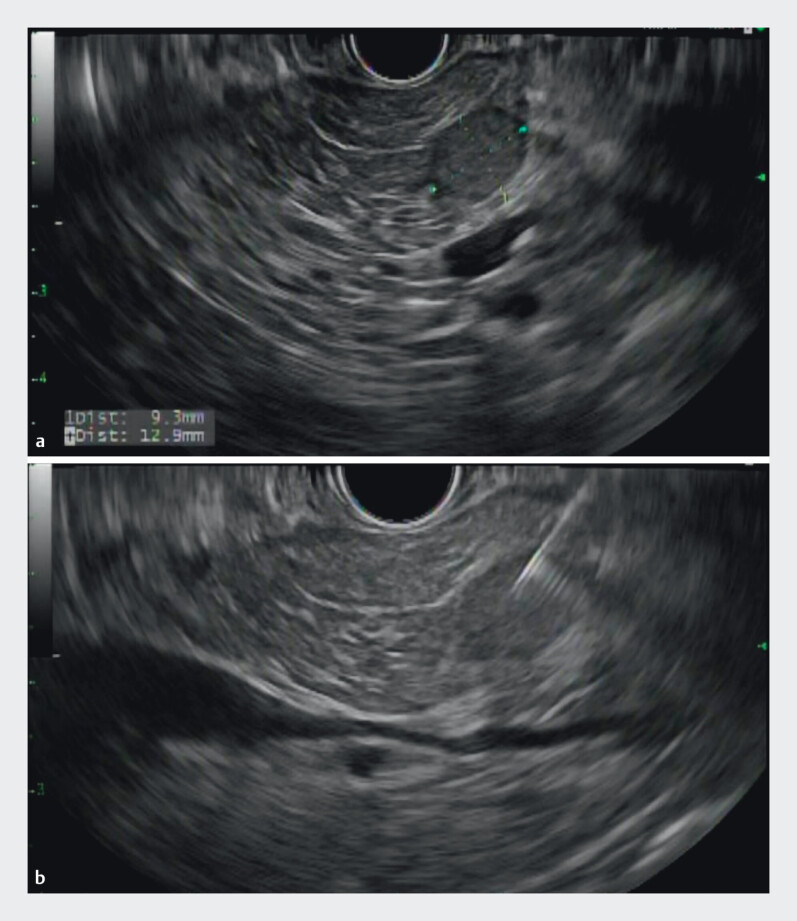
Endoscopic ultrasound (EUS) image showing: **a** the insulinoma in the pancreatic head; **b** EUS-guided fine-needle biopsy being performed.

This case highlights EUS-RFA as an effective minimally invasive option for insulinomas in select patients who are not ideal surgical candidates or who elect not to undergo surgery. Further prospective trials are needed to validate the long-term efficacy of EUS-RFA and better characterize its safety profile, including the incidence and management of potential adverse events.

Endoscopy_UCTN_Code_TTT_1AS_2AI

**Correction**

**Correction: Endoscopic ultrasound-guided radiofrequency ablation for a pancreatic insulinoma: a novel endoscopic management strategy** Ahmed Khalid, Karna Rahul, Alutaibi Mohammad et al. Endoscopic ultrasound-guided radiofrequency ablation for a pancreatic insulinoma: a novel endoscopic management strategy. Endoscopy 2025; 57: E1147–E1148, doi:10.1055/a-2712-3773 In the above-mentioned article the author name of Kidmealem Zekarias has been corrected. This was corrected in the online version on January 28, 2026.

## Notes

Endoscopy E-Videos https://eref.thieme.de/e-videos
*E-Videos* is an open access online section of the journal *Endoscopy* , reporting on interesting cases and new techniques in gastroenterological endoscopy. All papers include a high-quality video and are published with a Creative Commons CC-BY license. Endoscopy E-Videos qualify for HINARI discounts and waivers and eligibility is automatically checked during the submission process. We grant 100% waivers to articles whose corresponding authors are based in Group A countries and 50% waivers to those who are based in Group B countries as classified by Research4Life (see: https://www.research4life.org/access/eligibility/ ). This section has its own submission website at https://mc.manuscriptcentral.com/e-videos .

## Notes

Correction **Correction: Endoscopic ultrasound-guided radiofrequency ablation for a pancreatic insulinoma: a novel endoscopic management strategy** Ahmed Khalid, Karna Rahul, Alutaibi Mohammad et al. Endoscopic ultrasound-guided radiofrequency ablation for a pancreatic insulinoma: a novel endoscopic management strategy. Endoscopy 2025; 57: E1147–E1148, doi:10.1055/a-2712-3773 In the above-mentioned article the author name of Kidmealem Zekarias has been corrected. This was corrected in the online version on January 28, 2026.
