# Correction: Endoscopic ultrasound-guided radiofrequency ablation for a pancreatic insulinoma: a novel endoscopic management strategy

**DOI:** 10.1055/a-2797-6318

**Published:** 2026-01-28

**Authors:** Khalid Ahmed, Rahul Karna, Mohammad Alutaibi, Jimmie Stewart III, Kidmealem Zekarias, Guru Trikudanathan

**Affiliations:** 1 311816 Department of Medicine, Division of Gastroenterology, Hepatology and Nutrition, University of Minnesota System, Minneapolis, United States; 2 311816 Department of Laboratory Medicine and Pathology, University of Minnesota System, Minneapolis, United States; 3 311816 Department of Medicine, Division of Diabetes, Endocrinology and Metabolism, University of Minnesota System, Minneapolis, United States



In the above-mentioned article the author name of Kidmealem Zekarias has been corrected. This was corrected in the online version on January 28, 2026.

